# Antibiotic prophylaxis in shoulder arthroplasty — increased number of early infection revisions in patients treated with clindamycin compared to cefuroxime

**DOI:** 10.1016/j.jseint.2026.101774

**Published:** 2026-08-05

**Authors:** Jenni Harjula, Mari Kanerva, Juha Kukkonen, Kaisa Lehtimäki, Joel Kostensalo, The Finnish Shoulder Arthroplasty Registry Working Group, Pirjo Honkanen, Kaisa Lehtimäki, Katriina Paasikallio, Ville Ärimaa, Mikko Kahri, Tommi Viitanen, Mikko Salmela, Keijo Mäkelä, Jari Hartzell, Marko Vähäkuopus, Konsta Pamilo, Ville Äärimaa

**Affiliations:** a Department of Orthopedics and Traumatology, Turku University Hospital, Turku, Finland; b Department of Hospital Hygiene & Infection Control, Turku University Hospital, Turku, Finland; c Department of Orthopedics and Traumatology, Satakunta Central Hospital, Pori, Finland; d Natural Resources Institute Finland (LUKE), Joensuu, Finland

**Keywords:** Shoulder, Arthroplasty, Periprosthetic infection, Antibiotic prophylaxis, Clindamycin, Cefuroxime

## Abstract

**Background:**

Periprosthetic joint infection is a serious complication following shoulder arthroplasty. The purpose of this study was to compare the risk of early infection revision in patients given either clindamycin or cefuroxime antibiotic prophylaxis at time of surgery.

**Methods:**

We analyzed electronically recorded data from the Finnish Shoulder Arthroplasty Registry from 2016 to 2021. The primary end point was revision due to infection within 1 year after surgery. Hazard ratios (HRs) with 95% confidence intervals were calculated using Cox proportional hazards regression to compare clindamycin and cefuroxime prophylaxis. Age and sex were included as covariates in the model. Because revision indications were frequently missing, sensitivity analyses were conducted using predefined time-based scenarios.

**Results:**

There were 3,595 registered primary shoulder arthroplasties during the study period. Of these, 1,382 (38%) received clindamycin and 1,050 (29%) cefuroxime prophylaxis; prophylaxis information was missing for 969 (27%) arthroplasties. There were 87 (2.4%) revisions within 1 year (among these 12 (14%) were recorded as infection-related, and in 63 cases (76%), the reason for revision was missing). Six infection revisions occurred in the clindamycin group and 1 in the cefuroxime group. In the analysis including only confirmed infections, the adjusted HR for infection revision with clindamycin compared with cefuroxime was 4.51 (confidence interval 0.54-37.56; *P* = .16). In the sensitivity analyses accounting for missing revision indications, HR estimates ranged from 1.78 to 3.19, respectively. The absolute difference in 1-year infection-free survival between groups ranged from 0.3 to 0.9 percentage points, favoring cefuroxime.

**Conclusion:**

Clindamycin antibiotic prophylaxis may result in more early infection revisions in shoulder arthroplasty when compared to cefuroxime prophylaxis. This finding suggests that cefuroxime may be preferred over clindamycin for routine prophylaxis, although missing data warrant cautious interpretation.

Shoulder arthroplasty (SA) is a highly effective procedure for treating severe degenerative shoulder joint disease. Consequently, the number of SAs is rapidly increasing.^8^^,^^10^^,^^17^ As with other major joint arthroplasties, an important early complication of SA is periprosthetic joint infection (PJI), with a reported risk of 1–4%.^5^^,^^7^^,^^14^^,^^20^ Appropriate antibiotic prophylaxis at time of surgery is one method to reduce the risk of this serious complication.

The most common bacteria encountered in shoulder PJIs include *Cutibacterium acnes*, coagulase negative staphylococci, and *Staphylococcus aureus*.^21^ Clindamycin has been reported to be a generally effective antibiotic against these pathogens, and it has demonstrated persisting bioavailability with high levels of bone and joint penetration.^4^ Therefore, it has commonly been used as antibiotic prophylaxis in SA. However, clindamycin is primarily bacteriostatic, acting through binding to the 50S ribosomal subunit and inhibiting protein synthesis. In contrast, beta-lactam antibiotics, including cephalosporins, are inherently bactericidal against susceptible organisms.^13^ It is plausible that bactericidal antibiotics may provide more effective prophylaxis in preventing PJIs.^15^

The purpose of this study was to compare the incidence of early infection-related revisions in patients who received either clindamycin or cefuroxime prophylaxis for SA using data from the Finnish Shoulder Arthroplasty Registry (FAR). Our hypothesis was that cefuroxime prophylaxis would be associated with fewer early revisions due to infection than clindamycin prophylaxis.

## Materials and methods

FAR has collected data on shoulder arthroplasties since 1980, but because reporting before 2016 was paper-based and incomplete, we included only electronically reported data from January 2016 through December 2021. All hospitals and private clinics performing SA surgery in Finland are obligated to report patient-related data (gender, age, and diagnosis) and operative data (date, indication, arthroplasty type, and brand), both for primary and revision arthroplasties. Data on antibiotic prophylaxis (clindamycin, cefuroxime, vancomycin, other) at time of surgery are also recorded.

The primary end point was revision due to infection within 1 year after primary arthroplasty. Revision was defined as a removal, exchange, or addition of any prosthetic component. Because data on indication for revision were frequently missing, we also included patients with revision for unknown reason within predefined time frames (60 or 365 days) in the analyses. Each patient's unique civil registration number was used to link primary and revision procedures, and to track patients who had died. For patients who died during the study period the date of death was regarded as the end of follow-up.

### Statistical analysis

Descriptive statistics were calculated based on demographic data, arthroplasty type, follow-up time, and time to revision. Kaplan-Meier survival analysis was used to estimate infection-free survival within 1 year. Cox proportional hazards model^6^ was used to calculate the hazard ratios (HRs) with 95% confidence intervals (95% CI) comparing clindamycin and cefuroxime prophylaxis. Age (continuous variable) and sex were included as covariates in the model. Arthroplasty type was evaluated as a potential covariate; however, due to a substantial proportion of missing data and lack of improvement in model fit, it was not adjusted in the final adjusted model. Patients with bilateral SA procedures were included in the survival analysis as 2 separate observations, each shoulder treated as an independent procedure. The level of statistical significance was set at *P* < .05 and all tests were two-sided. Differences in the number of revisions between the years were assessed using Fisher exact test.

To address missing revision indications, 3 predefined sensitivity scenarios were analyzed:

1) Conservative scenario — only confirmed infections were considered events
2) Intermediate scenario — revisions with missing reason occurring within 60 days of the index surgery were considered infections
3) Extended scenario — all revisions with missing reason occurring within 365 days of index surgery were considered infections.

All analyses were done using the statistical software R (version 4.2.2).^16^

## Results

A total of 3,595 primary shoulder arthroplasties were recorded during the study period. There were 2,274 female and 1,321 male patients. The mean age at the primary surgery was 69 years (range 16–100 years). Arthroplasty types included 211 hemiarthroplasties, 889 total shoulder, and 1,121 reverse shoulder arthroplasties; arthroplasty type was missing in 1,374 cases (42%). Patient demographics are presented in Table I. One thousand three hundred eighty-two (38%) patients received clindamycin and 1,050 (29%) received cefuroxime as prophylaxis. For 969 (27%) patients, data on antibiotic were not available in the registry, and 4 patients did not receive any antibiotics (Table II).

**Table I:** Demographic details of the cohort.

Sex	Female: 2,274 (63%)	Male: 1,321 (37%)		
Age	Mean: 69	IQR: 13	Range: (16, 100)	
Side	Right: 2078 (58%)	Left: 1,491 (41%)	Both: 25 (1%)	Missing: 1 (0.0%)

*IQR*, interquartile range.

**Table II:** Antibiotic prophylaxis at time of primary surgery in different patient groups (% of patients within the prophylaxis group).

Antibiotic prophylaxis	N	Revisions			
		Infection	Other	Missing	Total
No	4	0	0	1	1
Cefuroxime	1,050	1	3	17	21
Cefur., clindam.	2	0	0	1	1
Cefur., other	2	0	0	1	1
Cefur., vancom.	5	0	1	0	1
Clindamycin	1,382	6	9	38	53
Clindam., other	8	0	1	0	1
Clindam., vancom.	3	1	0	0	1
Other	160	1	0	4	5
Vancomycin	10	0	0	1	1
Missing	969	0	0	17	17

*Cefur*, cefuroxime; *clindam*, clindamycin; *vancom*, vancomycin.

The overall number of revisions for any reason during the first year after index surgery was 87 (2.4%). The total number of revisions during the study period was 121 (2.9%). There was no statistically significant difference in the annual number of revisions across the study years (*P* = .95). Among the 87 early revisions, 12 were recorded as revisions for infection (14%), 3 (3%) for instability, and 6 (7%) for other reasons, and in 63 (76%) cases, the reason for revision was missing. In the clindamycin group, 6 confirmed early infection revisions occurred; revision indication was missing in 38 additional cases. In the cefuroxime group, 1 confirmed early infection revision occurred; revision indication was missing in 17 cases. The median time for reported early infection revision was 28 days (standard deviation 25, mean 36 days).

**Figure 1:**
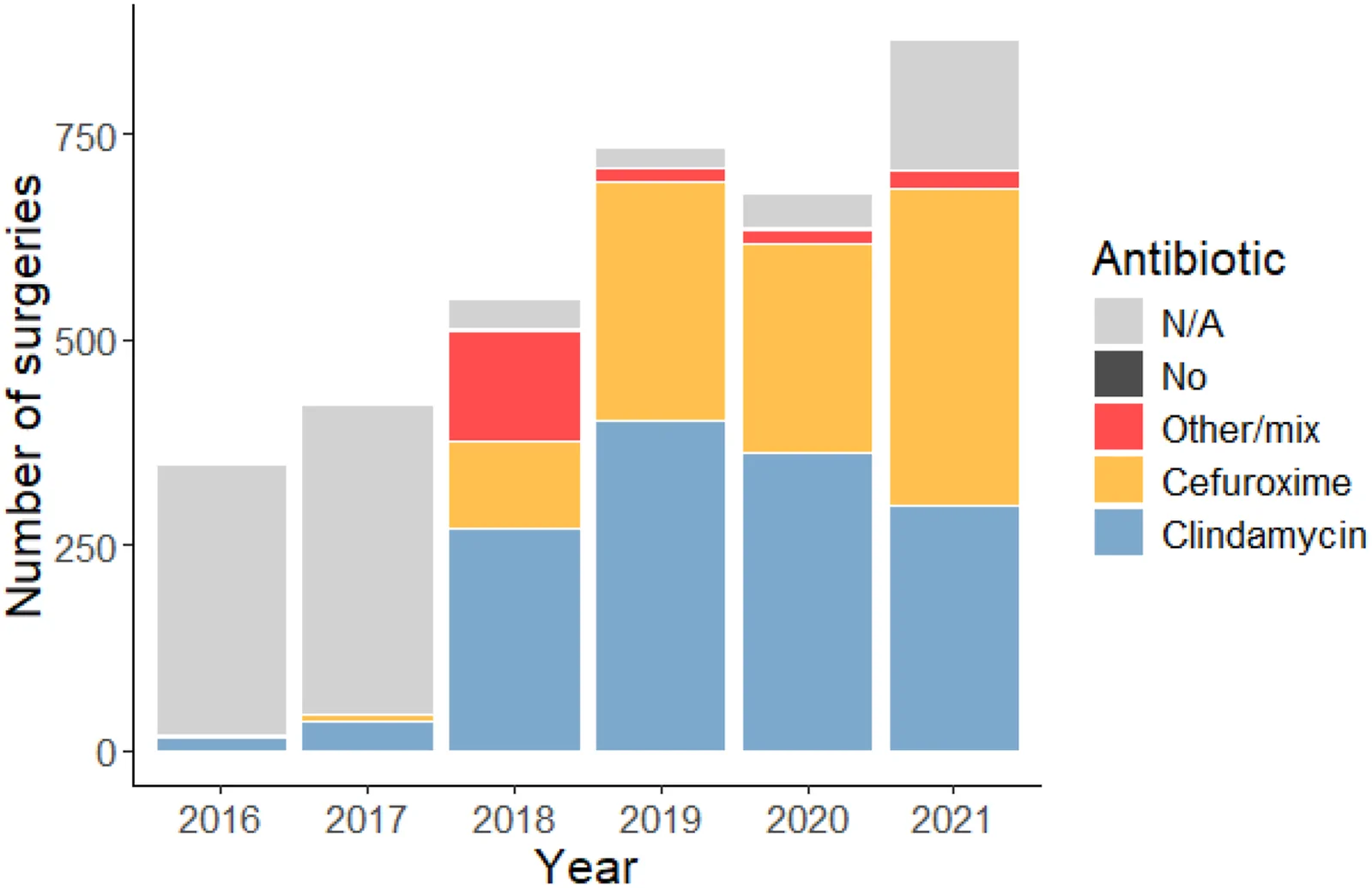
The medication used for antibiotic prophylaxis over the study period. *N/A*, not applicable.

The use of antibiotic prophylaxis as a function of time is shown in Figure 1. Seventy-three percent of the missing prophylaxis data originated from 2016 to 2017. Clindamycin was more frequently used in the early years of the observation period, whereas cefuroxime use increased over time and surpassed clindamycin in 2021. The use of other antibiotics or combinations remained low throughout the later study years.

**Figure 2:**
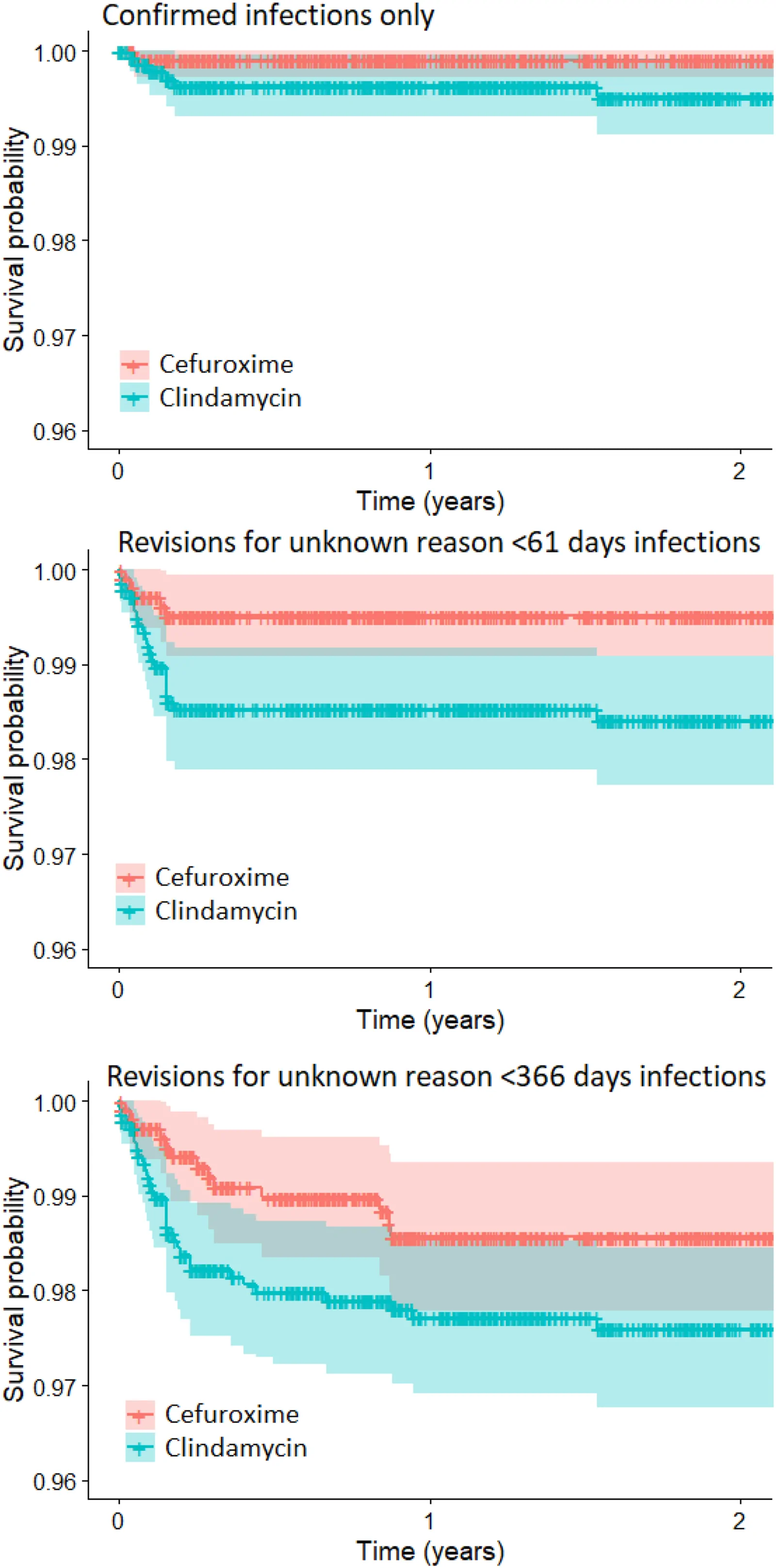
The survival curves for revision due to infection for the 2 main antibiotics in 3 different scenarios for the missing data: confirmed infections only, missing reason within 60 days assumed infection, missing reason within 365 days assumed infection.

Kaplan-Meier estimates of 1-year infection-free survival and adjusted HRs from Cox regression models are presented in Table III. Kaplan-Meier survival curves for the 3 predefined missing data scenarios are shown in Figure 2. In all cases infection-related revision was considered the event of interest.

**Table III:** The Kaplan-Meier estimates for survival without infection at 1-year postsurgery for patients receiving cefuroxime or clindamycin and the corresponding hazard ratio for clindamycin compared with cefuroxime.

Scenario	Kaplan-Meier at 1 y		Cox	
Missing revisions	Cefuroxime	Clindamycin	Odds ratio (clind.)	*P* value
Excluded	0.999 [0.997, 1.000]	0.996 [0.993, 1.000]	4.51 [0.54, 37.56]	.16
<61 d infections	0.995 [0.991, 0.999]	0.985 [0.979, 0.992]	3.19 [1.20, 8.47]	.02
<366 d infections	0.986 [0.978, 0.994]	0.977 [0.969, 0.985]	1.78 [0.93, 3.41]	.08

*clind*, clindamycin.

Results for different scenarios for missing revisions are given in the first column.

### Scenario analysis

In the conservative (complete-case) analysis including only confirmed infections, the survival probability at 1 year was 0.996 (95% CI [0.993, 1.000]) for clindamycin and 0.999 (95% CI [0.997, 1.000]) for cefuroxime. The adjusted HR for clindamycin compared with cefuroxime was 4.51 (95% CI [0.54, 37.56], *P* = .16).

In the intermediate (within 60 days) scenario, the corresponding numbers were 0.985 (95% CI [0.979, 0.992]) for clindamycin and 0.995 (95% CI [0.991, 0.999]) for cefuroxime. The adjusted HR was 3.19 (95% CI [1.20, 8.47], *P* = .02).

In the extended (within 365 days) scenario, these numbers were 0.977 (95% CI [0.969, 0.985]) for clindamycin and 0.986 (95% CI [0.978, 0.994]) for cefuroxime. The adjusted HR was 1.78 (95% CI [0.93, 3.41], *P* = .08).

Additional variations of the sensitivity analyses are provided in the Supplementary Material and yielded results consistent with the intermediate (within 60 days) scenario.

In the 3 scenarios, neither sex nor age was associated with early infection risk (*P* = .07–.70). Arthroplasty type could not be fitted in the model due to large number of missing data.

## Discussion

The main finding of this nationwide registry study was that clindamycin prophylaxis was associated with a higher risk of early infection revision compared with cefuroxime prophylaxis after SA.

Prophylactic recommendations for SA have largely been extrapolated from hip and knee arthroplasty, where antibiotic prophylaxis has been shown to reduce infection risk substantially.^1^ In knee replacement surgery, Robertsson et al^19^ (2017) reported a higher risk of revision for infection in patients receiving clindamycin compared with cloxacillin. Data specifically addressing antibiotic prophylaxis in SA remain limited. A previous registry study suggested differences in infection reoperation rates using combination of cloxacillin and benzylpenicillin prophylaxis versus clindamycin alone.^7^ In accordance with our findings, Marigi et al^11^ and Nieboer et al^12^ reported a lower rate of PJI with cefazolin prophylaxis than with clindamycin.

Cephalosporins inhibit bacterial cell wall synthesis and are bactericidal against susceptible organisms. Cefazolin is a first-generation cephalosporin, whereas cefuroxime is a second-generation drug with a wide target spectrum. On the contrary, clindamycin is mainly bacteriostatic, and from a theoretical perspective, bactericidal agents may be preferable for surgical prophylaxis. Clindamycin use has also been associated with an increased risk of *Clostridioides difficile* infection.^9^ In addition, antimicrobial resistance patterns may also influence prophylactic effectiveness, and emerging resistance of *C. acnes* to clindamycin has been reported.^2^ While susceptibility testing specific to cefuroxime is not routinely reported in Finland, available data suggest that *C. acnes* strains remain susceptible to beta-lactam antibiotics.^24^^,^^25^ It is noteworthy that both cefuroxime and clindamycin may cause hypersensitivity reactions. Cefuroxime allergy is less common (<0.001%) than, for example, reported penicillin allergy and most patients with reported penicillin allergy tolerate cefuroxime.^23^

Infection risk after SA is multifactorial. Surgical factors such as draping, operative technique, and duration may influence infection risk.^18^^,^^20^ However, patient-related factors (eg, obesity, smoking, hyperglycaemia, immunosuppressive states) are also important contributors to infection risk.^18^^,^^20^ As the indications for SA get wider, the risk of infection is also likely to increase. Especially patient's young age and male sex have previously been reported as key risk factors for PJI after SA.^18^^,^^20^ In our study, analyses were adjusted for age and sex. Other potential confounders could not be accounted for due to sparsity of registry data.

The effectiveness of antibiotic prophylaxis also depends on appropriate dosing and timing. American Academy of Orthopaedic Surgeons guidelines recommend the administration of antibiotic prophylaxis within 1 h of the surgical procedure with the recommended dosage of, for example, 1.5 g of cefuroxime or 600–900 mg of clindamycin.^3^ Re-dosing is recommended during operation after 2 half-lives of the antibiotic (T_1/2_ for cefuroxime is 1–2 hours and for clindamycin 2-4 hours) or if there is blood loss exceeding 1,500 ml.^22^ Unfortunately, our data do not allow for further analyses on the dosing details.

This study has several limitations. First, the registry-based observational design limits control over several confounding variables. Second, we do not have data on reporting completeness of FAR, and it is likely that there were missing cases in the early study period. Third, indication for revision was missing in a substantial proportion of reported cases, and we performed predefined sensitivity analyses to assess this potential impact. The number of confirmed infection revisions was also low and resulted in wide confidence intervals. Fourth, the data are based solely on registry reporting by the surgeon. Therefore, we do not have, for example, microbiological data on the pathogen. Despite these limitations, the study has strengths, including nationwide coverage, mandatory reporting, and the ability to analyze rare adverse events in a large cohort. The overall 1-year revision rate observed in our cohort (2.4%) is within the range reported by large national arthroplasty registries, supporting the external validity of our findings. The consistent direction of association across sensitivity analyses suggests that the observed difference may be clinically relevant, although confirmation in studies with more detailed clinical and microbiological data is warranted.

## Conclusion

In this nationwide registry study, clindamycin prophylaxis was associated with a higher risk of early infection revision compared with cefuroxime. However, given the low number of confirmed infections and the proportion of missing data, these findings should be interpreted with caution. Further studies are needed to corroborate these results and especially to optimize the dosing and type of antibiotics used as prophylaxis in SA.
