# Supplementary material for: Antibiotic prophylaxis in shoulder arthroplasty — increased number of early infection revisions in patients treated with clindamycin compared to cefuroxime
Source: JSES Int. 2026 Aug 5;10(6):101774. doi: 10.1016/j.jseint.2026.101774 (PMC13571292; doi:10.1016/j.jseint.2026.101774)
Supplement: Supplementary file 1 [file mmc1.docx]

**Supplementary material to ANTIBIOTIC PROPHYLAXIS IN SHOULDER ARTHROPLASTY – increased number of early infection revisions with clindamycin compared to cefuroxime**

**Sensitivity analysis with more scenarios**

While three scenarios corresponding to conservative, realistic, and extreme scenarios for missing revisions were discussed in detail in the paper, additional analyses were done to ensure that the conclusions are robust. In total, eight different scenarios were considered: 1) confirmed infections only 2) any revision without a reason available which happen in the <31 days is assumed to be an infection 3) same but <61 days 4) same but <101 days 5) same but <366 days 6) multiple imputation based on the distribution of reasons over time 7) the <61 day scenario but missing prophylaxis cases are clindamycin 8) multiple imputation but the missing prophylaxis cases are clindamycin.

The known infection cases happened mostly early, with a median of 30 days. The three infection cases happening the latest were at 66, 95, and 562 days, respectively. The choice of 30, 60, and 100 days was based on these numbers, as 30 days is the median, 60 days covered 75% of the cases, and 100 days all but the one outlier. In the multiple imputation scenario, 100 imputation rounds were carried out and the results pooled. The data were divided into two epochs with 80.5 days (i.e., average of 66 and 95 days) as a dividing line. In these two epochs, the fraction of infection revisions of all known revisions was calculated for both clindamycin and cefuroxime separately. Then the missing revision reasons were randomly assigned as an infection or other with the corresponding probabilities. The missing prophylaxis data were mostly from the years 2016 and 2017 and at this time clindamycin was much more popular, and thus the missing cases would be more likely to be clindamycin.

The results are given in Table S1. The results from the additional scenarios are closest to the “realistic” scenario (<61 days) discussed in the article.

**Table S1.** The Kaplan-Meier estimates for survival without infection at one-year post surgery for patients receiving cefuroxime or clindamycin and the corresponding odds ratio for choosing clindamycin over cefuroxime. Results for seven different scenarios for missing data are listed with either data with a missing medication excluded or assumed to be clindamycin, and missing revision reasons either imputed or excluded from the analysis.

| **Scenario** | | **Kaplan-Meier at 1 year** | | **Cox** | |
| --- | --- | --- | --- | --- | --- |
| **Missing**  **antibiotic** | **Missing revisions** | **Cefuroxime** | **Clindamycin** | **Hazard ratio (clind.)** | **p-value** |
| Excluded | Excluded | 0.999 [0.997, 1.000] | 0.996 [0.993, 1.000] | 4.51 [0.54, 37.56] | 0.16 |
| Excluded | <31 days infections | 0.997 [0.994, 1.000] | 0.991 [0.986, 0.996] | 3.31 [0.94, 11.63] | 0.06 |
| Excluded | <61 days infections | 0.995 [0.991, 0.999] | 0.985 [0.979, 0.992] | 3.19 [1.20, 8.47] | 0.02 |
| Excluded | <101 days infections | 0.993 [0.988, 0.998] | 0.982 [0.975, 0.989] | 2.50 [1.13, 5.50] | 0.02 |
| Excluded | <366 days infections | 0.986 [0.978, 0.994] | 0.977 [0.969, 0.985] | 1.78 [0.93, 3.41] | 0.08 |
| Excluded | Multiple imputation | 0.994 [0.989, 0.999] | 0.983 [0.976, 0.990] | 3.09 [1.27, 7.54] | 0.01 |
| Clindamycin | <61 days infections | 0.995 [0.991, 0.999] | 0.988 [0.983, 0.992] | 2.61 [1.01, 6.77] | 0.05 |
| Clindamycin | Multiple imputation | 0.994 [0.989, 0.999] | 0.987 [0.982, 0.991] | 2.54 [1.07, 6.06] | 0.04 |
